# Why don't Chinese college students seek help from the National Health Service (NHS)? Chinese college students' use of medical services in the UK

**DOI:** 10.1016/j.heliyon.2024.e37879

**Published:** 2024-09-12

**Authors:** Zheng Yang, Yuanting Huang

**Affiliations:** The School of Communication, Soochow University, China

**Keywords:** Medical services, Intercultural environment, Chinese students, Sick role, Illness experience

## Abstract

**Background:**

International students have a lower utilization rate of the local medical service system for studying abroad, and it has been found that there may be multiple reasons behind this phenomenon. This study explores the usage of medical service systems by international students and the underlying logical factors through a study of the usage of National Health Service (NHS) of Chinese students in the UK.

**Methods:**

To address the research questions, this study employed an online survey methodology that ran between 1st May and August 20, 2019 facing the Chinese students in the UK. A total of 1,050 questionnaires were distributed and 1,001 questionnaires were recovered, of which 977 contained valid responses (questionnaire response rate was 95.3 % and validity rate was 97.6 %). Before the questionnaire was designed and after it was issued, two focus group interviews were conducted to provide reliable and detailed information to inform the questionnaire design and to supplement the questionnaire survey data with more profound psychological qualitative data. The two focus groups consisted of 10 and 12 Chinese students studying in the UK and each lasted more than 3 h.

**Results:**

The survey data showed that the medical services utilization rate of Chinese students in the UK is relatively low compared to UK residents and domestic Chinese students. Their decisions and behaviours around medical services usage in the UK are not significantly related to age, gender, and monthly income, but are instead related to their current education status, types of disease suffered, and information acquisition about the UK medical services before coming to the UK. When getting sick, in addition to seeking help from official medical services, Chinese students studying in the UK tend to self-diagnose and self-medicate; seeking help from social networks based on friendship and domestic relatives are also alternatives to accessing medical services.

**Conclusion:**

Combining the theories of ‘sick role’ and ‘illness experience’, the decisions and behaviours related to medical services usage by Chinese students in the UK are significantly influenced by their understanding of medical services, which is socially and culturally learned in China. Understanding the perspective of the ‘sick role’ and the ‘illness experience’ of Chinese students may help to better think about how improvements can be made to their utilization rate of medical services and their health status during their studies in the UK. This study not only provides us with specific information and understanding on the usage of medical services for Chinese students in the UK, but the research results may also provide a reference for other similar research on the health and medical service use of other international students studying in the cross-cultural contexts.

## Highlights

• Chinese students' medical services utilization rate is relatively low in the UK.
• Chinese students' medical services usage behaviour is related to their education status, disease suffered, and information acquisition.
• Official medical service is not the first choice of Chinese student to deal with disease in the UK.
• Cognitive framework hinders Chinese students' effective use of medical services in the UK.

## 1 Introduction

### 1.1 Description of the context

The health status and medical services use of groups living in a cross-cultural environment has long been a key issue in the study of sociology of medicine and health [[1], [2], [3]]. Among those groups, the medical and health status of international students studying overseas became a social issue that many countries had to address, especially following the global spread of Covid-19 in 2020 [4,5]. Different cultural backgrounds, barriers to language communication, and other institutional factors mean that groups who live in a cross-cultural environment, such as international students, experience difficulties in seeking medical services and thus have a low utilization rate of official medical services [2,6,7].

According to the data released by the Ministry of Education of China, the number of Chinese students studying abroad in 2019 totalled 703,500, making them an important social group that cannot be ignored in Chinese society [8]. Within this figure, students studying in the UK accounted for 25 % of the total, second only to the United States [8]. In July 2019, the UK Student Health Association together with the Chinese Students and Scholars Association UK (CSSAUK) held a seminar on the medical services use and health status of Chinese students studying in the UK, which shows that the medical services usage of Chinese students has begun to be taken seriously in both China and the UK. However there is currently no clear data and research available in relation to the medical services usage of Chinese students in the UK. Therefore, this study mainly focuses on the investigation and analysis of Chinese students' use of medical services in the UK and its corresponding influencing factors, through a quantitative UK-wide survey. The results of this study may provide a better understanding of the medical service usage of groups in a cross-cultural environment, including international students, and indicate how to promote the medical service usage of these groups to improve their health status.

Medical or health services refers to a public service system providing any medical or remedial care or service. In the UK, it is shorthand for the National Health Service (NHS) [9], the UK's publicly funded health care system. NHS and private treatments are the two main channels for UK residents, as well as international students studying the UK, to seek medical help. Among them, the official medical system represented by the NHS occupies the main part [9]. Studies have already shown that the usage of medical services by individuals or specific groups is jointly affected by multiple internal and external factors, such as their demographic variables: gender, age, educational level, income and so on [[10], [11], [12], [13], [14]], and also the external cultural environment and social system [2]. These factors are also the focus of this article analyzing the medical service usage of Chinese students in the UK. Understanding the usage of Chinese students' medical services in the UK can not only help the UK universities and other relevant government departments to better understand the health statues and behavioural characteristics of Chinese students in the UK, but also help to provide targeted suggestions for the UK medical service system and agencies, the UK Students' Federation, the UK Student Health Association, the UK Chinese Students' Federation, and other Chinese governments or relevant organizations, such as Chinese Service Center for Scholarly Exchange (CSCSE) to work together to improve quality of Chinese students' health and their medical services usage in the UK. Therefore, this research is not only about Chinese students themselves and their parents, but the research results are also of great significance to the university, related governments and organizations, and other stakeholders.

### 1.2 Theoretical background

Medical services usage is considered to profoundly and obviously affect an individual's health status [2]. Similarly, a group's medical services usage and its tendency will also affect that group's overall health status. For instance, Jetty et al. (2021) found that, due to the inability to adapt to the mainstream medical system, the utilization rate and usage effect of medical services by minority populations in the US after illness was lower than that of White Americans [15]. This lower utilization rate is further reflected in the relatively undesirable health situation of Mexican Americans. The decisions and behaviour around medical services usage of an individual or group are considered to be affected by various factors, as a result of the complicated interactions between the individual/groups and the medical service system [16,17]. Therefore, exploring the significant factors that affect the decisions or behaviour of medical services usage of an individual or a group can effectively help to identify how to improve the health status of that individual or group [1,2].

Many studies have found that demographic differences profoundly affect the decisions and behaviours of medical services usage of different groups/individuals, such as age [2,10]; gender [10,11,13,18]; income [[19], [20], [21]]; social class [3,14,19,22]; education level [12,23]; and nationality [24,25]. For instance, Phelan and colleagues found that the difference in mortality between different social classes in the United States is largely determined by the differences in the attitudes and use behaviours of different social classes towards medical services [26]. Data from National Center for Health Statistics also shows that women's utilization rate of medical services is much higher than men. In addition to demographic factors, Geertsen and other scholars also found that one's social networks and social interactions also significantly affect decisions related to medical services use. Structural institutional factors – whether residents can easily access and pay for medical services, including early medical system registration and later medical treatment – are also considered to be important factors affecting use of medical services, combining demographics factors such as race, income, and social class [3,14,19,22,27]. In addition, language and cultural differences have also been found to have a profound impact on behaviour around medical services usage of individuals/groups, especially in a cross-cultural environment [28,29] (Adato et al., 2011; Kwame, 2016). For patients with different cultural backgrounds, such as international student studying abroad, it may be difficult for them to interact with medical services providers smoothly, which affects their experience of using medical services for the first time, and in turn affects their subsequent medical service use decisions when they fall ill [1]. For instance, Eisenberg and colleagues (2011) found that compared with native students in the United States, the utilization rate of mental health services for international students is much lower [30]. Ellis-Bosold and Thornton-Orr (2013) also found barriers for Chinese international students in using available health services on American university campuses [31]. And during COVID-19 pandemic, Van de Velde et al. (2021) also found that international students have worse performance in response to the epidemic than local students [32].

However, on the one hand, as Cockerham (2010; 2017) observed, sociodemographic variables can only explain whether there are differences in the use of medical services among different groups – they cannot explain why there are such differences [1,2]. Furthermore, the use of medical services is not only affected by the sociodemographic variables of those users, but also restricted by the external socio-cultural environment and social system of medical services [2], especially under the cross-cultural background, the influence of this kind of cultural environment and social system becomes more obvious [30,31]. Under the combined influence of such multiple internal and external factors, even similar groups, such as students, often show different mechanisms of medical services usage under different backgrounds and situations.

It is just because the use of medical services is not only affected by demographic factors that can be easily measured by survey or other quantitative methods, but also by cultural and behavioural factors that are subtle and difficult to quantify. Therefore, to further explore what affects the behaviour of specific groups in their use of medical services, more detailed qualitative explanations need to be sought based on personal behaviour at a psychological level. On the other hand, the use of medical services by international students from various countries differs. For instance, Russell et al. (2008) found that international students were always under-utilizing both health and counselling services [33]. Skromanies et al. (2018) believe that it is because that international students always lack accessible, targeted, and culturally sensitive health information support [34]. Although there have already been some studies focusing on the health behaviour of international students [35,36], it is still under-researched of exploring the medical service use decisions and behaviours of a particular group of international students, especially treating international students as a non-homogenous mass. Therefore, in relation to a particular individual or group, such as Chinese students in the UK, it is necessary to examine the specific context from which they come more detail in order a more precise explanation for their behaviour in relation to medical services [1,2], and to conduct a detailed empirical analysis from both qualitative and quantitative perspectives. Such research results can not only improve the effectiveness of medical services for Chinese students in the UK, but also have certain reference significance for other international student groups in other countries, especially by comparing this research conclusion with the research findings from other countries [[33], [34], [35], [36]].

To better guide the study, the following two research questions are addressed in this paper:

RQ 1 What is medical services usage like for Chinese students in the UK?

RQ 2 What factors affect the decisions and behaviour around medical service use of Chinese students in the UK?

## 2 Methods

### 2.1 Data collection

To address the two research questions, this study employed an online survey methodology that ran between 1st May and August 20, 2019. A total of 1,050 questionnaires were distributed and 1,001 questionnaires were recovered, of which 977 contained valid responses (questionnaire response rate was 95.3 % and validity rate was 97.6 %). The issuance of the online questionnaire is mainly dependent on the Chinese Student Federation of the UK universities to reach as many and diverse groups of Chinese students as possible. Although this kind of sampling method that relies on the Chinese Student Federation may have a certain sample bias, it can indeed help us obtain more effective Chinese student samples.

The survey questionnaire was composed of six parts and 24 questions: demographic questions, such as gender, age, educational levels, monthly disposable income; sources of information about the UK medical service system for Chinese students; basic situation of Chinese students using medical services in the UK; motivation for Chinese students to use medical services in the UK; Chinese students' experience of using medical services in the UK; and Chinese students' perceptions of the differences between Chinese and UK medical services. The design of the questionnaire mainly refers to Van Den Brink et al.‘s research [37] and the Health Care Utilizaton Reports and Survey Instructions published by the US State Health Planning & Development Agency [38], and some specific issues and options that Chinese students are concerned about found in the first focus group have also been added. The questionnaire was distributed by the Chinese online questionnaire software Sojump and entrusted to the All-China Students' Federation of 34 universities in the UK to distribute.

Before the questionnaire was designed and after it was issued, the research team conducted two focus group interviews. The first aimed to provide reliable and detailed information to inform the questionnaire design [39], such as identifying detailed and accurate channels for Chinese students in obtaining UK medical service system information before arriving in the UK, and the main reasons why students might use medical services during their study abroad. The second focus group interview was mainly used to supplement the questionnaire survey data with more profound psychological qualitative data. The two focus groups consisted of 10 and 12 Chinese students studying in the UK and each lasted more than 3 h. The participants of focus group interviews rely on snowballing methods and are recruited according to demographic differences (such as age, gender, stage of study) in The University of Sheffield with the help of the Chinese Students’ Association of The University of Sheffield. Two focus group interviews were both semi-structured. For those interviewees who had never used medical services during their studies in the UK, the interviews and discussion was more about the reasons for their refusal to do so, and the alternative sources they adopted. With the consent of all interviewees, the two sessions were recorded and later transcribed for further in-depth analysis.

### 2.2 Statistical analysis

The whole questionnaire survey participants included Chinese students at 34 UK universities distributed across 21 UK cities (n = 977); 53.4 % were female and 44.4 % male (2.2 % preferred not to report their gender). The average age was 23.6 (SD = 6.78). Most participants were masters (n = 313, 32 %), undergraduate (n = 254, 26 %), and pre-masters students (n = 166, 17 %). PhD (n = 127, 13 %), while pre-undergraduate (n = 88, 9 %) and exchange students (n = 29, 3 %) made up the remaining groups of participants. All questionnaire data will be first subjected to descriptive analysis, followed by regression analysis using SPSS 24.0 with NHS as the independent variable. For focused group interview data, the method of qualitative text analysis based on the grounded method was launched to discover as many factors as possible that influence Chinese students to adopt or avoid UK medical services.

### 2.3 Trustworthiness

Through the Alpha reliability test and KMO validity test via SPSS 24.0, the average reliability value of the variables in the questionnaire was 0.91 and the average construct validity value was 0.82, which are both within acceptable range.

## 3 Findings

### 3.1 Basic situation of the medical service usage of Chinese students in the UK

Before exploring the factors that influence the medical services usage for Chinese students in the UK, it is necessary to investigate the basic situation to answer the first research question and provide a snapshot of medical services usage. The analysis in this section is mainly based on survey data.

According to the survey, only 47 % of respondents (n = 977) had used official UK medical services in the past year. But 89 % of respondents believed that they had had more than once situation that needed medical services or official medical help in the past year (Table 1). The large gap between existing needs and actual use of medical services shows that Chinese students in the UK do have a low medical utilization rate after self-accepting the ‘sick role’. According to the UK Medical Service Utilization survey by NatCen, 98 % of UK adult residents have registered for the NHS and use it and other medical services at least once a year [40,41]. According to another survey, the medical service utilization rate of domestic Chinese college students in different provinces has remained above 70 % (such as Shandong, 76.2 %; and Anhui, 74.8 %) [42,43]. The large difference in the utilization rate of medical services of Chinese students in the UK compared to domestic Chinese students and local residents in the UK also shows how low the medical service utilization rate of Chinese students in the UK really is, and this is especially clear in the field of mental health. Among the respondents, 56 % believed that they had experienced mental illness which needed professional psychological intervention or treatment during their studies in the UK, but only 34 % had actually sought such treatment (Table 1).

**Table 1:** Medical services utilization rate of Chinese students in the UK.

	Yes	No
Have used medical services in the past year	47 %	53 %
Have needed to use medical services in the past year	89 %	11 %
Have used mental health services in the past year	34 %	66 %
Have needed to use mental health services in the past year	56 %	44 %

The survey results further show that using UK official medical services is not the first choice of Chinese students after getting sick in the UK, instead they tend to opt for ‘Self-diagnosis and self-medication’. Contacting domestic relatives and friends for help and seeking help from classmates, roommates and other friends are also important options for Chinese students after getting sick in the UK (Table 2).

**Table 2:** First choice of Chinese students when getting sick in the UK.

Q:When you get ill or need medical help while studying in the UK, what is your first choice?	
Self-diagnosis and self-medication	45 %
Seek help from official medical services, such as NHS or GP	26 %
Contact domestic relatives and friends for help	16 %
Seek help from classmates, roommates and other friends in the UK	12 %
Other	1 %

Traditional medical sociology believes that self-diagnosis, self-medication, and self-care are often applicable when a person is familiar with one's own symptoms, and the required treatment methods and possible results are already known [1,44]. Stevenson and others also pointed out that even self-diagnosis does not mean complete independence from medical professional help such as purchasing drugs and conducting physical examinations that also require interaction with medical professional services [45]. However, through focus group interviews, it was found that Chinese students in the UK are almost completely separated from UK medical professional services when conducting self-diagnosis (the reasons for this are explained below). One Chinese student observed:

I have almost never bought medicines in the UK. Basically, I brought all kinds of medicines I need from China. Even if I want to buy some anti-fever and painkillers or sugar for pharyngitis at Tesco or Boots, I just check it online by myself first, and then go to Boots to find it directly. I hardly communicate with shop assistants. (No. 15)

Intra-group subsidies, or seeking help based on interpersonal relationship are also common alternative measures for Chinese students facing illness in the UK, such as:

When I was sick in the UK, I often called my parents back home as soon as possible. They were very familiar with my body and were able to provide many useful suggestions based on the situation I described. (No.8)

I usually seek help from my classmates or friends around me in the UK. On the one hand, I know that they always brought a lot of medicine from China. On the other hand, some of my friends have medical backgrounds. I trust them very much. (No.11)

### 3.2 Demographic factors and medical services usage of Chinese students in the UK

As discussed above, several empirical studies have shown that demographic factors such as age, gender, education level, and income are significant factors affecting residents' use of medical services. For instance, women and the elderly are generally considered to be more inclined to use medical services after getting ill [1,10,11,18,46], people with higher education level and higher disposable income are also considered more inclined to use medical services [3,14,19,20,22]. Therefore, we proposed the first hypothesis: H1: demographic variables can predict the healthcare system usage behaviour of Chinese students in the UK.

However, in this study, through a logistic regression likelihood ratio test via SPSS 26.0, there was found to be no significant correlation between age, gender, and monthly disposable income as independent variables and whether or not Chinese students in the UK use medical services and their first behaviour choices after illness as dependent variables. But there is a significant correlation between the behavioural choices of Chinese students after getting ill with their educational level (Table 3). Chinese students studying in the UK at different stages of education (pre-undergraduate, undergraduate, pre-masters, masters, pre-doctoral, doctoral, and postdoctoral) have different choices in coping behaviours after illness. Through a further linear regression analysis of the educational status of Chinese students in the UK and the frequency of medical service use, there is a significant positive correlation between the two (Table 3): the higher the degree being studied by Chinese students in the UK, the more inclined they are to use official medical services after illness. The insignificance of age and monthly disposable income may be related to the small difference in age and monthly disposable income among Chinese students studying in the UK.

**Table 3:** Relationship between demographic factors and medical services usage of Chinese students in the UK.

Dependent variable:Whether Chinese students use medical services					
Effect	Model Fitting Criteria	Likelihood Ratio Tests			
	−2 Log likelihood	Chi-square	df	Sig.	
Intercept	734.222a	.000	0	.	
Age	737.666	3.444	7	.841	
Sexuality	736.060	1.838	2	.399	
Education	784.761	50.539	11	.000	
Income	739.518	5.296	5	.381	
Dependent variable: The choices Chinese students make when they are sick					
Intercept	1711.376a	.000	0	.	
Age	1750.289	38.913	35	.298	
Sexuality	1715.397	4.021	10	.946	
Education	1811.078	99.702	55	.000	
Income	1724.935	13.559	25	.969	
Dependent variable: Frequency of Chinese students' medical usage					
Model	B	Std. Error	Standardized Coefficients	t	Sig.
Intercept	−.037	.192		−4.888	.000
Education	.145	.032	.142	4.547	.000

### 3.3 Other influencing factors on medical services usage of Chinese students in the UK

Through the focus group interview conducted before the survey, it was found that the type of diseases suffered, and a person's understanding of UK medical services may affect decisions and behaviours of medical services usage in the UK. Therefore, we proposed the second and third hypothesis: H2: the types of diseases experienced can predict the healthcare system usage behaviour of Chinese students in the UK; and H3: the information acquisition about NHS can predict the healthcare system usage behaviour of Chinese students in the UK.

To verify these possible correlations, the types of diseases experienced and whether information was obtained about UK medical services through certain channels before coming to the UK were set as independent variables, while use of medical services in the UK was set as the dependent variable for correlation analysis. It was found that in the past year, Chinese students in the UK who suffered from traumatic diseases such as fractures, sudden diseases such as acute gastroenteritis, and infectious diseases such as flu were more inclined to use official medical services, while daily diseases such as colds or coughs, chronic diseases such as chronic gastritis, and major diseases like heart disease did not show an obvious correlation with Chinese students' decisions and behaviours of medical services usage in the UK (Table 4). In the survey, 55 %, 51 % and 46 % of respondents also said that they would seek help from the UK medical service system because of sudden illnesses, traumatic diseases, and infectious diseases, respectively. In addition, whether or not they had been exposed to relevant information about UK medical services before coming to the UK also affected use of medical services for Chinese students studying in the UK (Table 5): Chinese students who had received information about the UK medical services system before going abroad preferred to use the medical service system in the UK during studies when they were sick. However, statistics also show that information about UK medical services from different channels had varied influences on Chinese students' decisions around medical services usage in the UK. Through the first focus group interview, a list of channels was drawn up relating to those which Chinese students might use to obtain information about UK medical services before arriving in the UK. These channels were set as independent variables to examine the relationship with Chinese students' decisions around medical services usage in the UK. The statistics show that only two information channels, ‘pre-departure training sessions held by Chinese universities and schools’ and ‘digital media accounts of the UK All-China Students’ Federation (ACSF), such as WeChat or Weibo’, had a significant impact on such behavioural inclinations of Chinese students during their studies in the UK.

**Table 4:** Relationship between disease types and medical services usage of Chinese students in the UK.

Dependent variable: Whether Chinese students use medical services				
Correlation			df	Sig.
Independent variables	Daily diseases (e.g. cold)	.160	1	.689
	Traumatic diseases (e.g. fracture)	.705	1	.001
	Sudden diseases (e.g. acute gastroenteritis)	.728	1	.000
	Chronic diseases (e.g. chronic gastritis)	1.644	1	.200
	Major diseases (e.g. heart disease)	.002	1	.962
	Infectious diseases (e.g. flu)	.837	1	.000
	Others	3.434	1	.064
		7.51	7	.262

**Table 5:** Relationship between information channels and medical services usage of Chinese students in the UK.

Coefficients^a^							
Control Variables	Model		Understandardized	Coefficients	Standardized	t	Sig.
			B	Std. Error	Coefficients Beta		
Gender, age, educational level and monthly disposable income	1	(Constant)	.659	.095		6.904	.000
		Information acquisition	.332	.035	.311	9.574	.000
Gender, age, educational level and monthly disposable income	1	UK universities websites	−.014	.043	−.010	−.326	.745
		Emails from UK universities	.037	.042	.026	.868	.386
		Pre-departure training sessions	.045	.042	.110	3.543	.000
		Digital media accounts of ACSF	.179	.045	.119	3.963	.000
		Commercial digital accounts	−.017	.046	−.011	−.362	.717
		Study abroad agencies	.049	.055	.027	.899	.369
		Friends	−.056	.058	−.029	−.060	.333
		Others	−.044	.086	−.016	−.514	.608

a Dependent Variable: whether students had used medical services in the UK.

Summarizing the data analysis of the above three section, the utilization rate of medical services of Chinese students in the UK is relatively low, and it is not affected by gender, age, and monthly disposable income, but to a certain extent it is related to students’ current education status and the types of diseases suffered during their study period. Their decisions and behaviours around medical services usage in the UK are also significantly affected by their information acquisition about UK medical services before coming to the UK, and different information channels could have different effects. However, the above results cannot effectively explain why the medical service utilization rate of Chinese students in the UK is much lower than that of local residents in the UK and Chinese domestic college students. This issue is addressed in the subsequent quantitative and qualitative analysis.

### 3.4 Factors hindering the medical services usage of Chinese students in the UK

The survey data shows that across all respondents, whether they had used medical services in the UK before or not, agreed that the cumbersome medical treatment process, language barriers, and high time costs were the top three significant factors that hindered Chinese students’ medical services usage in the UK (Table 6).

**Table 6:** Main reasons hindering Chinese students in using UK medical services.

Question: Which factors do you think are the main reasons hindering you in using UK medical services?				
Ranking	All respondents (n = 977)		Respondents who had never used medical services in the UK (n = 518)	
1	Cumbersome process of medical services	78 %	Cumbersome process of medical services	85 %
2	Language disability	54.%	Language disability	64 %
3	Time cost	52 %	Time cost	48 %
4	Having self-diagnosis ability	34 %	Economic cost	43 %
5	Economic cost	25 %	Having self-diagnosis ability	33 %
6	Other	8 %	Other	3 %

The cumbersome medical services process was considered by the Chinese students in the UK as the most important factor hindering their use of medical services. When international students are sick, they are asked to contact their GP first, then make an appointment for a face-to-face consultation, and then proceed with further treatment or transfer to the hospital based on the GP's diagnosis and recommendations. Compared with the medical procedures in China, those in the UK are not simple, but neither or they very complicated. The source of the fear of cumbersome medical treatment will be analysed in the subsequent discussion section, although such fear relies on the social networks of Chinese students to spread. A postgraduate in the focus group said:

Before I came to the UK, I heard from my seniors that it is very troublesome to see a doctor in the UK, and it is even more difficult for you to go to the hospital by yourself. So, they suggested me to bring as much medicines as possible from China, and then try to take them by myself when I get sick in the UK. (No. 4)

The fear of a cumbersome medical services process is also reflected in the respondents' different perceptions of Chinese and UK medical services. Among all respondents, 80.4 % thought that it was easier to seek medical treatment in China, and only 17 % it was easier in the UK. The fear of cumbersome medical procedures in the UK is further related to the excessively high cost of medical treatment in Chinese students’ understanding. One interviewee in the focus group said:

In China, when I am unwell, I just go to the hospital to register, queue up and see a doctor. The whole journey takes only half a day. But in the UK, I need to make an appointment first, and then wait for an appointment. It often takes me 2–3 days to see a doctor. (No.5)

In addition to the cumbersome process of medical services and high time cost, language barriers are also a significant factor hindering Chinese students from seeking medical services in the UK. In the traditional view of medical sociology, insufficient language communication skills of patients often directly lead to inadequate or even fractious communication between doctors and patients, further leading to misjudgement and misdiagnosis of diseases [47](Berkanovic, 1980). Since doctor-patient communication relies heavily on medical language, it is more difficult for international students in a cross-language environment. At the same time, language communication barriers not only affect the diagnosis and treatment of patients' diseases, but also profoundly affect patients' feelings about the medical services process and influence their subsequent medical behaviours by influencing their experience of first medical services use. For instance, one Chinese doctoral student said:

When I first arrived in the UK, I once felt uncomfortable and called the NHS. But because my English is really bad, I simply don't understand what they are talking about. In the end, I made many phone calls, but little helped. Later, I went to see my GP, and I found that on the one hand, I couldn't describe my symptoms effectively, and I didn't understand the medical words the GP said. Therefore, I didn’t want to see the doctor anymore in the UK. (No.13)

In addition to the above factors, the results from survey also show that although all Chinese students are required to purchase immigration health insurance (the Immigration Health Surcharge, IHS) when applying for a visa before going to the UK, to be submitted to the Visa Centre as an important attachment in the student visa application process – which means that all Chinese students in the UK can use official medical services for free – Table 6 shows, 25 % of all respondents and 42.74 % of respondents who had not used medical services believed that excessive medical service costs were an important reason hindering their use of medical services in the UK. On the one hand, this the shows that IHS and other related institutions have not effectively communicated information about medical service guarantees and expenses in the UK to the Chinese students studying there. On the other hand, it may also relate to the cognitive habits of Chinese students regarding medical services. In the second focus group interview, one pre-masters student said:

Really? Is it really free? I don't know it at all. You all know how much it costs to see a doctor in China. My goodness. I have never heard of it. I thought it would be very expensive. (No.3)

In summary, the survey and focus group interview show that, for Chinese students in the UK, the cumbersome process of medical services, the excessively high cost of time, language barriers, and the misunderstanding of medical expenses due to a lack of correct information are the main reasons Chinese students are unwilling to actively use medical services when they are sick in the UK. The cumbersome medical treatment process, language barriers, and high time costs are the top three most significant factors that hinder Chinese students’ medical services usage in the UK.

## 4 Discussion

### 4.1 Help-seeking, sociocultural mediation, and cognitive framework

Based on the findings above, Chinese students are unwilling to actively use medical services when they are sick in the UK. In fact, this phenomenon does not only exist in the group of Chinese students in the UK. According to Russell et al. and Perry et al., international students also were always under-utilizing both health and counselling service in Australia and USA [33,48]. Some existing studies have also attempted to analyze this phenomenon and point out that lack of accessible, targeted, and culturally sensitive health information support; students' health-related experiences, the health support provided by the universities and even students' personal, academic, and post-graduation aspirations are all related to whether they choose to use the medical service system [[34], [35], [36]]. But the influencing factors that these findings focus on are relatively scattered and diverse, lacking a core theoretical integration. Facing this, this study attempts to answer the question of why international students are unwilling to use the local official medical service system, starting from the theory of ‘sick role’.

Parsons pointed out that people's acceptance and understanding of the ‘sick role’ is affected by individual, environmental and social understanding [49,50]. Among these, individual understanding mainly focuses on a person's own perception of the sensory function and its abnormality; environmental understanding is biased towards the understanding of whether self-sensory abnormality is ‘deviant’ in a person's surroundings; while social understanding relates to a person's decision-making cognition of follow-up behaviour on the basis of ‘deviance’. Based on the research into sick roles and illness experience, Mechanic further suggested that although individual help-seeking behaviour is affected by multiple cognitive levels, both the individual and the environmental cognition level are culturally and socially learned [51]. Mechanic's ‘help-seeking theory’ believes that no matter what kind of cognition the individual's help-seeking behaviour is based on, it needs to complete the ‘other-defined’ and ‘self-defined’ process of disease and sick role. The ‘other-defined’ is the important prerequisite for individuals to obtain the socialized sick role; while ‘self-defined’ is the important basis for individuals to decide what kind of help-seeking behaviour to use in their ‘sick role’. An individual's understanding of their own health state, sick role, and disease process is mediated by their social and cultural background. Sociocultural background plays a huge role in shaping the individual's understanding and response to diseases [52]. Individuals living in a cross-cultural environment usually also graft the cognitive framework they have learned from their original sociocultural background to the cross-cultural environment in which they are living [[51], [52], [53]]. This explains why Chinese students in the UK believe that the costs of medical services in the UK are too high without obtaining relevant information about the UK medical system. In the Chinese context, medical services have always been regarded as a major expense. This kind of understanding has been further amplified and strengthened by the media, and there have even been various reports of selling estate for getting medical services in China. Chinese students acquire an understanding of the high cost of medical services in the Chinese sociocultural environment. And without fully understanding the UK medical service system, they easily graft such a cognitive framework onto the UK medical service system, which leads to a misunderstanding about the medical services in the UK and impacts their decision to refuse to use such services.

In addition, the medical services usage habits acquired by Chinese students from many years in China will also affect their perception, evaluation and final usage of the UK medical process. When individuals experience something that is different or contrary to their original cognitive framework, they tend to make more negative evaluations of such things, and further avoid making choices that violate their self-cognition framework [54,55]. Therefore, when Chinese students in the UK learn that the medical services process is different from the one, they are used to in China – either through experience or rumour – they may tend to avoid choosing to use such services, and instead choose to self-diagnose and self-medicate with domestic drugs that they are already familiar with. This ‘help-seeking theory’ also explains to a certain extent why more than 80 % of the respondents believe that it is more convenient to seek medical treatment in China than in the UK, even though half of these respondents had not used UK medical services.

The cognitional and behavioural biases based on the learned framework from the original sociocultural environment can often be broken by further information acquisition [56,57]. When people know more about new things, their existing cognitive framework has less influence [57,58]. This also explains why Chinese students' decision and behaviour around medical service use in the UK has a significant relationship with their information acquisition of UK medical services. Thus, to break Chinese students’ prejudiced understanding of the UK medical services caused by their learned cognitive framework from China, it is necessary to carry out in-depth and detailed education of those students before and after they go abroad.

### 4.2 Sick role, illness experience, and medical services usage

**Fig. 1:**
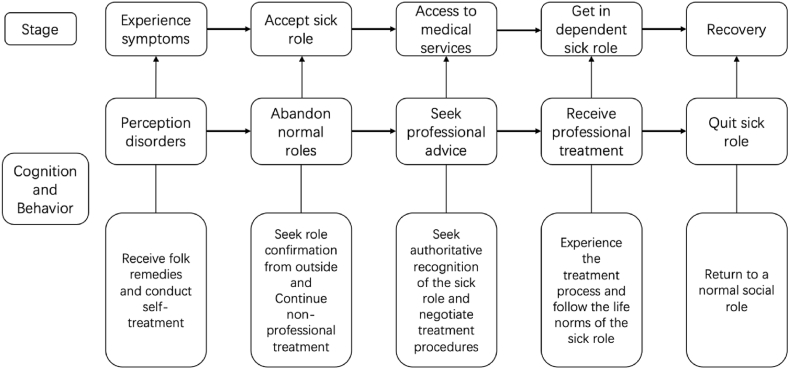
Illness experience process model (Suchman, 1965; Coe, 1970; 1978).

According to the ‘illness experience process model’ proposed by Suchman [59], when an individual thinks they are sick, they will go through five different reaction stages based on their specific illness experience: 1) experience symptoms; 2) accept sick role; 3) access medical services; 4) assume dependent sick role; and 5) recovery. Coe further explored the different cognitions, decisions and behaviours of individuals at different stages [60,61] (Fig. 1). According to Suchman and Coe, personal illness experience begins when symptoms first appear. At this stage, the individual's perceptions begin to change, causing them to make a decision about whether to pay attention to symptoms based on their understanding of such abnormality and whether to treat the symptoms as a health problem. Meanwhile, individuals often rely on their own lay knowledge system and use folk remedies and self-care to treat themselves. In the Chinese context, one of the most representative cases, facing this, is the advice to ‘drink more hot water when you feel uncomfortable’ [62]. Once the individuals define their abnormality as a disease, they enter the ‘sick role’ state [59]. Therefore, individuals in ‘sick role’ are allowed to shed their normal social responsibilities. Although the individual's self-treatment and folk remedies continue, the individual is forced to decide whether or not to seek professional medical help. If the individual accepts the ‘sick role’ on their own and starts seeking medical professional help, then they enter the third stage of medical care contact. At this stage, the individual hopes to obtain a ‘legitimate’ sick role recognized by the authority and to negotiate with this authority, such as doctors, regarding the treatment process. If both the individual and the authority agree on the ‘sick role’ and the necessity of treatment, the individuals will have a phased dependence on medical services and enter the role of dependent patient until recovery. However, for some chronic diseases, even if the patient feels good about themselves and has given up the ‘sick role’ and assumes social responsibility again, their role in the fourth stage may continue [1,2,59].

For Chinese students in the UK, especially those who thought they needed medical help in the past year, they had already completed the first and second stages of their illness experience. In the focus group interviews, almost all the interviewees when talking about their illness experience said that whether they sought help from the medical services or chose self-medication, they all slowed down or suspended their learning tasks, and even asked for leave when they felt sick. For the students, this meant suspending their social responsibility. Although this temporary suspension was sometimes ‘illegal’, it also indicates that they had officially abandoned the normal social role and accepted the sick role. For those Chinese students who were unwilling to use UK medical services, in accepting their sick role, their illness experience is suspended in the third stage. To understand why so many Chinese students suspended in the third stage of the illness experience, the benefits of entering the third stage and the corresponding influencing factors in this stage need to be analysed.

**Fig. 2:**
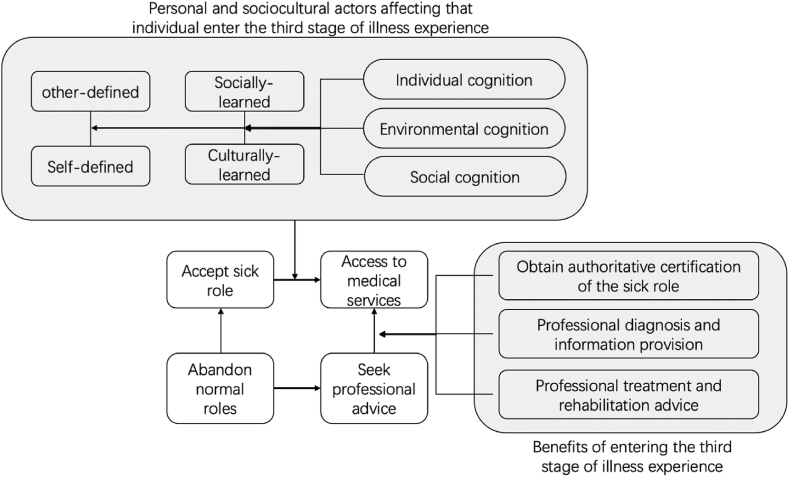
Factors affecting individual illness experience.

According to Suchman and Coe, when an individual enters the third stage of illness experience, they can gain benefits from three levels: first, they can obtain authoritative certification of the ‘sick role’ from the professional medical service system – their ‘sick role’ can be ‘legalized’, allowing them to gain a legitimate exemption from their original social responsibility [[59], [60], [61]]. In addition, seeking professional advice in the third stage can also help patients gain professional knowledge of their illness; and further seek professional treatment based on such knowledge [59,61,63]. Parsons also pointed out that in terms of understanding of the ‘sick role’ and illness experience, no matter which stage the individual is in, or from which stage they transition, they are all affected by their individual, environmental and sociocultural understanding, especially when the function of modern medicine is regarded as a method of social control [49,50]. Therefore, combined with the discussion above, the factors that affect Chinese students in the UK in effectively transitioning from the second to the third stage when they have medical service needs can be summarized in the following model (Fig. 2).

In terms of the benefits that can be provided in the third stage, although professional medical services can provide patients with professional diagnosis, medical information, and further professional treatment and rehabilitation advice, these benefits all need to be established based on adequate doctor-patient communication [63,64] (Cockerham et al., 1986; Rier, 2000). The adequacy of doctor-patient communication is affected by cultural and language factors [1,2,65,66]. Chinese students who lack professional English medical language skills as patients generally lack the ability to effectively interact with local doctors in the UK. There is also a huge social distance between Chinese student patients and UK local doctors who belong to different ethnic and social cultural backgrounds in terms of doctor-patient communication [[67], [68], [69]], and such social distance also results in the inability to exchange sufficient information and knowledge between the two groups. This makes it difficult for Chinese student patients to effectively obtain the benefits provided by entering the third stage of the illness experience process. And as mentioned above, alternative measures based on intra-group subsidies and personal social interactions and communication between Chinese students domestically and abroad make it easier for Chinese students to obtain medical support and help in other ways, especially in a cultural and language familiar interaction environment. In addition, the importance of the authoritative certification of the ‘sick role’ and the exemption from social responsibility are related to the social roles assumed by the individual and the degree of the Bindingness of Social Responsibility (BCR) of such social roles [70]. The lower the BCR of one's social role, the less important the authoritative certification of the ‘sick role’ and the exemption from social responsibility will be. Compared with Chinese domestic school management, UK universities are more relaxed in the daily management of students [71]. Student groups have relatively fewer social responsibility behaviours based on student status than they have in China. This has led to a lower degree of BCR for Chinese students studying in the UK. Therefore, compared with domestic Chinese students, Chinese students in the UK are more likely to directly choose to skip classes and other behaviours that violate the social responsibility of students [71]. Therefore, in terms of the benefits that can be provided by entering the third stage of the illness experience, the degree of beneficial results brought about by the authoritative certification of the patient's role is reduced, due to the low BCR of Chinese students in the UK; the beneficial results of professional diagnosis and treatment are likely to be weakened by insufficient patient-doctor communication. Compared with UK local residents and domestic Chinese students entering the third stage of the illness experience process, it is difficult for Chinese students in the UK to obtain equal benefits. Thus, compared with those two groups, Chinese students have a lower official medical services utilization rate. The above analysis shows that both ‘help-seeking theory’ and ‘illness experience’ theory are effective in explaining the use of medical services by Chinese students studying in the UK, but the certain elasticity in explaining and the reasons that Chinese students do not seek help from the UK official medical system also show that the particularity of Chinese students and the Chinese culture behind it makes the application of ‘help-seeking theory’ and ‘illness experience’ theory should also consider the particularity of social culture and the group. These theories can also effectively integrate various reasons why international students are unwilling to use the local medical service system, such as lack of health information support or expectations for their academic aspirations [[34], [35], [36]](Skromanies, 2018; Rogowsky et al., 2020; Sanci et al., 2022).

## 5 Conclusion

This study first presented basic information related to the medical services usage of Chinese students in the UK. The survey data showed that the medical services utilization rate of Chinese students in the UK is relatively low compared to UK residents and domestic Chinese students. Their decisions and behaviours around medical services usage in the UK are not significantly related to age, gender, and monthly income, but are instead related to their current education status, types of disease suffered, and information acquisition about the UK medical services before coming to the UK. When getting sick, in addition to seeking help from official medical services, Chinese students studying in the UK tend to self-diagnose and self-medicate; seeking help from social networks based on friendship and domestic relatives are also alternatives to accessing medical services. Based on the theories of illness experience and sick role, the illness experience process of Chinese students who do not prefer to use the UK official medical services is suspended at the transition from the second to the third stage. This suspension is largely influenced by the cognitive framework of Chinese students gained from their experience of medical services in China. Understanding the perspective of the ‘sick role’ and the ‘illness experience’ of Chinese students may help to better think about how improvements can be made to their utilization rate of medical services and their health status during their studies in the UK. In order to effectively improve the utilization rate of medical services of Chinese students in the UK, on the one hand, it can work with the Chinese government to strengthen information training for those international students before going abroad, and enhance their familiarity with the medical service process in the UK; on the other hand, UK medical services institutions such as the NHS should also collaborate with the UK universities to strengthen training on cross-cultural medical communication for those international students and correct the misunderstandings caused by cross-cultural factors. Simultaneously improving the service process efficiency and service speed of medical services institutions such as the NHS is also an effective way to further enhance the utilization rate of official medical services for Chinese international students in the UK. This study not only provides us with specific information and understanding on the usage of medical services for Chinese students in the UK, but the research results may also provide a reference for other similar research on the health and medical service use of other international students studying in the cross-cultural contexts. There are also some limitations of this study, all data used in the study were collected before the COVID-19 epidemic. The global epidemic of COVID-19 may change the health behaviour of overseas students. This issue requires further exploration and analysis in our subsequent research. And using the students from only one university as the focus group interview sample also have some bias, which needs to expand the sample range in the subsequent research.

## Data availability statement

The original contributions presented in the study are included in the article/Supplementary Materials, further inquiries can be directed to the corresponding author.

## Ethical statement

Ethical Community:

Ethics Administrator office of Sociological Studies.

The University of Sheffield.

Data of approval: 23/11/2018.

Approval Number: 023021.

## CRediT authorship contribution statement

**Zheng Yang:** Writing – review & editing, Supervision, Methodology, Formal analysis, Conceptualization. **Yuanting Huang:** Writing – review & editing, Software.

## Declaration of competing interest

The authors declare that they have no known competing financial interests or personal relationships that could have appeared to influence the work reported in this paper.
