# Supplementary material for: Why don't Chinese college students seek help from the National Health Service (NHS)? Chinese college students' use of medical services in the UK
Source: Heliyon. 2024 Sep 12;10(18):e37879. doi: 10.1016/j.heliyon.2024.e37879 (PMC11417328; doi:10.1016/j.heliyon.2024.e37879)
Supplement: Multimedia component 1 [file mmc1.docx]

**Questionnaire on the use of NHS by Chinese students in the UK**

1. What is your age?

2. What is your gender?

A. Male

B. Female

C. Prefer not to say

3. Your current educational status in the UK:

A. About to start a foundation year for undergraduate studies in the UK

B. Currently undertaking a foundation year for undergraduate studies in the UK

C. About to start undergraduate studies in the UK

D. Currently undertaking undergraduate studies in the UK

E. About to start a foundation year for master's studies in the UK

F. Currently undertaking a foundation year for master's studies in the UK

G. About to start master's studies in the UK

H. Currently undertaking master's studies in the UK

I. About to start doctoral studies in the UK

J. Currently undertaking doctoral studies in the UK

K. Other

4. Your monthly disposable income in the UK is approximately:

A. Below £800

B. £800-£1200

C. £1200-£1600

D. £1600-£2000

E. Above £2000

5. Did you have any knowledge about the school hospital, NHS, or other medical facilities in the UK before coming here?

A. Yes

B. No

5a. How did you primarily obtain information about the school hospital, NHS, or other medical facilities in the UK before coming here? (Multiple choices allowed)

A. Official school website

B. Official school emails

C. Official WeChat, Weibo accounts of Chinese student unions, etc.

D. WeChat, Weibo accounts related to studying abroad

E. Study abroad agencies

F. Recommendations from friends

G. Other

6. Did you consider any specific medical situations that might require local medical attention in the UK before coming here? (Multiple choices allowed)

A. No need for local medical attention

B. Common illnesses like cold, fever, etc.

C. Injuries like sprains, fractures, etc.

D. Acute conditions like gastroenteritis, etc.

E. Chronic conditions like gastritis, etc.

F. Major conditions like heart disease, etc.

G. Infectious diseases like viral flu, etc.

H. Other

7. Before coming to the UK, what kind of services did you hope the school hospital, NHS, or other medical facilities would provide? (Multiple choices allowed)

A. Diagnosis and treatment of common illnesses

B. Information and measures for disease prevention

C. Rehabilitation

D. Routine check-ups

E. Health advice and counseling

F. Other

8. Do you already have experience studying in the UK or have you started your studies in the UK?

A. Yes (Continue with the survey)

B. No (End of survey)

9. Have you ever sought medical attention at the school hospital, NHS, or other medical facilities while studying in the UK?

A. Yes

B. No

10. When you fell ill during your studies in the UK, what was your first choice?

A. Contacting a GP for medical assistance

B. Visiting a medical facility (including private medical facilities)

C. Self-diagnosis and self-medication

D. Seeking help from friends nearby

E. Other

11. What kind of illness do you think would lead you to seek medical attention at the school hospital, NHS, or other medical facilities?

A. Common illnesses like cold, fever, etc.

B. Injuries like sprains, fractures, etc.

C. Acute conditions like gastroenteritis, etc.

D. Chronic conditions like gastritis, etc.

E. Major conditions like heart disease, etc.

F. Infectious diseases like viral flu, etc.

G. Other

12. How often do you typically seek medical attention at the school hospital, NHS, or other medical facilities in the UK?

A. Once a month or more

B. Once every one to three months

C. Approximately every six months

D. Once a year

E. Once every one to two years

F. Once every two years or more

13. How long does a typical visit to the school hospital, NHS, or other medical facilities in the UK last?

A. Less than 15 minutes

B. 15-30 minutes

C. 30-60 minutes

D. More than an hour

14. Are you satisfied with your overall experience of seeking medical attention at the school hospital, NHS, or other medical facilities in the UK?

A. Very satisfied

B. Quite satisfied

C. Average

D. Quite dissatisfied

E. Very dissatisfied

15. Do you feel that your communication with the doctor during your medical visits is sufficient?

A. Very sufficient

B. Quite sufficient

C. Average

D. Quite insufficient

E. Very insufficient

16. Are you satisfied with the diagnosis provided by the doctor during your medical visits?

A. Very satisfied

B. Quite satisfied

C. Average

D. Quite dissatisfied

E. Very dissatisfied

17. Are you satisfied with the final treatment or handling of your condition by the doctor during your medical visits?

A. Very satisfied

B. Quite satisfied

C. Average

D. Quite dissatisfied

E. Very dissatisfied

18. What do you think are the main reasons hindering you from choosing to seek medical attention at the school hospital, NHS, or other medical facilities? (Multiple choices allowed)

A. Language barriers

B. Time and effort

C. Medical expenses

D. Complicated medical processes

E. Self-belief in being able to handle the illness

F. Other

19. What do you think are the main reasons encouraging you to seek medical attention at the school hospital, NHS, or other medical facilities? (Multiple choices allowed)

A. Severity of the illness

B. Impact of the illness on daily life

C. Inability to judge the illness by oneself

D. Other

20. Have you sought any psychological information or treatment in the UK?

A. Yes

B. No

21. Do you think you have experienced situations in the UK that required psychological counseling or treatment?

A. Yes

B. No

22. If there is a need for psychological counseling or treatment, what is the situation? (Multiple choices allowed)

A. Insomnia

B. Depression

C. Anxiety

D. Adjustment disorders

E. Delusions

F. Obsessive-compulsive disorder

G. Schizophrenia

H. Nervous breakdown

I. Other

23. Which do you think is more convenient to seek medical treatment in China or in the UK?

A. China

B. The UK

24. How do you think seeking medical attention at the school hospital, NHS, or other medical facilities in the UK differs from seeking medical attention in your home country? (Multiple choices allowed)

A. Medical processes

B. Doctor-patient communication

C. Physical examination processes

D. Diagnosis processes

E. Treatment processes

F. Access to medication

G. Other

25. Do you have any suggestions or comments on the medical environment in the UK? (Open-ended question)
